# Elevated cerebrospinal fluid uric acid during relapse of neuromyelitis optica spectrum disorders

**DOI:** 10.1002/brb3.584

**Published:** 2016-10-21

**Authors:** Yaqing Shu, Haiyan Li, Lei Zhang, Yuge Wang, Youming Long, Rui Li, Wei Qiu, Zhengqi Lu, Xueqiang Hu, Fuhua Peng

**Affiliations:** ^1^ Department of NeurologyThe Third Affiliated Hospital of Sun Yat‐sen UniversityGuangzhouChina; ^2^ Department of NeurologyThe Fifth Affiliated Hospital of Sun Yat‐sen UniversityZhuhaiChina; ^3^ Department of NeurologySecond Affiliated Hospital of Guangzhou Medical UniversityGuangzhouChina

**Keywords:** metabolism, excitotoxicity, neuroimmunology

## Abstract

**Introduction:**

Previous studies have shown that serum uric acid (UA) modulates outcomes of neurological diseases, although little is known about cerebrospinal fluid (CSF) UA levels in neuromyelitis optica spectrum disorders (NMOSDs).

**Methods:**

Cerebrospinal fluid and serum UA levels were measured in samples from 68 patients, including NMOSDs during relapse (*n *= 38) and controls with noninflammatory and non‐neurodegenerative diseases (CTLs, *n *= 30). Correlation analysis was performed between CSF UA and clinical characteristics, serum UA, and blood–brain barrier integrity in NMOSDs.

**Results:**

Cerebrospinal fluid UA levels in NMOSDs were significantly higher than in CTLs (*p *=* *.002), while serum UA differences between NMOSDs and CTLs were not statistically significant. In NMOSDs, CSF UA levels were significantly higher in patients with an impaired blood–brain barrier than in patients with an intact one (*p *<* *.001), and significantly higher in longer disease duration than in shorter disease duration patients (*p *=* *.002). CSF UA levels were also significantly higher in active patients upon MRI than in inactive patients (*p *<* *.001), and significantly higher in patients with brain lesions than without brain lesions (*p *=* *.024). CSF UA was significantly associated with the serum UA levels (*r *= .454, *p *=* *.002), disease duration (*r *= .383, *p* = .018), and blood–brain barrier index (*r *= .805, *p *<* *.001), but did not correlate with age, gender, annualized relapse rate, duration, or severity of NMOSD. Multiple regression analysis demonstrated that CSF UA was independent of the blood–brain barrier index (β = .765, *p *<* *.001) and serum UA levels (β = .01, *p *=* *.019) in NMOSDs.

**Conclusions:**

Cerebrospinal fluid UA levels were elevated in NMOSD patients during relapse, and were likely modified by serum UA levels and blood–brain barrier integrity.

## 1 Introduction

Uric acid (UA) is a natural product of the purine metabolic pathway and is a principal endogenous danger signal released from injured cells (Rock, Hearn, Chen, & Shi, 2005; Shi, Evans, & Rock, 2003). The role of UA in the central nervous system (CNS) remains poorly understood. It not only activates immune effectors in the innate and adaptive immune systems (Shi et al., 2003), but also enhances antibody immunity (Behrens et al., 2008). Some studies have also suggested that UA is a strong peroxynitrite scavenger and antioxidant (Hooper et al., 2000; Sevanian, Davies, & Hochstein, 1991; Waugh, 2008), while others have shown that UA reflects xanthine oxidase activity with its subsequent production of free radicals (Kanemitsu et al., 1989) and is associated with oxidative stress and glutamate‐mediated excitotoxicity in neurological patients (Stover, Lowitzsch, & Kempski, 1997).

Neuromyelitis optic (NMO) and multiple sclerosis (MS) are typical inflammatory demyelinating diseases of the CNS. Neuromyelitis optica spectrum disorders (NMOSDs) comprise NMO, Asian optic‐spinal MS, optic neuritis or longitudinally extensive myelitis associated with systemic autoimmune disease, optic neuritis or myelitis associated with brain lesions typical of NMO (hypothalamic, corpus callosal, periventricular, or brainstem), and limited forms of NMO (idiopathic single or recurrent events of longitudinally extensive myelitis and recurrent or simultaneous bilateral optic neuritis) (Wingerchuk, Lennon, Lucchinetti, Pittock, & Weinshenker, 2007; Wingerchuk, Lennon, Pittock, Lucchinetti, & Weinshenker, 2006). Oxidative stress is also thought to be involved in NMO and MS (Gonsette, 2008; Haider, 2015; Lassmann & van Horssen, 2016; Penton‐Rol et al., 2009). Previous studies have focused more on serum UA in NMO/MS patients (Ashtari, Bahar, Aghaei, & Zahed, 2013; Liu et al., 2013; Min et al., 2012; Peng et al., 2008; Sotgiu et al., 2002), although cerebrospinal fluid (CSF) UA levels might more directly reflect CNS. However, very little is known about CSF UA levels in NMOSDs. Therefore, we performed a hospitalized‐based study to investigate CSF UA levels in NMOSD patients and analyzed the correlation between CSF UA levels and clinical characteristics involving disease duration, disease disability assessed by the Expanded Disability Status Scale (EDSS) score (Kurtzke, 1983), and blood–brain barrier (BBB) integrity.

## 2 Methods

### 2.1 Ethics statement

This research was approved by the ethics committee of the Third Affiliated Hospital of Sun Yat‐sen University (No. 2007‐33) and the procedures followed were in accordance with the Helsinki Declaration of 1975, as revised in 2008. All participants involved in this study provided written informed consent.

### 2.2 Patients and methods

Our study recruited 68 patients, including 38 NMOSD patients (21 NMO, 6 recurrent optic neuritis, 7 Asian optic‐spinal MS, 2 optic neuritis associated with systemic autoimmune disease, 2 optic neuritis associated with brain lesions typical of neuromyelitis optica) and controls (CTLs, *n *= 30). All patients had been hospitalized during clinical acute relapse one or more time from 2007 to 2012. The NMOSDs had been diagnosed according to the criteria described previously (Wingerchuk et al., 2007). Patients enrolled in the database were diagnosed by at least two specialized neurologists. Those with noninflammatory and non‐neurodegenerative diseases (*n *= 30; 17 migraine, 13 lumbar back pain) were enrolled as controls (CTLs) during the same period. All samples were collected prior to treatment. None of the patients had been treated with acetylsalicylic acid, thiazide diuretics, steroids, or other drugs that could affect UA levels within the previous 12 weeks, and none of the patients were diagnosed with diabetes mellitus, renal disorders, hepatic disorders, or malignancies.

Demographic and clinical characteristics of patients are summarized in Table 1.

**Table 1:** Study population characteristics

	NMOSDs	CTLs
Number of patients	38	30
Gender (female:male)	29:9	17:13
Age at onset (years)	38.2 (17–65)	36.7 (6–76)
Disease duration (months)	23.9 (0.3–192)	N/A
ARR	0.8 (0.25–3.5)	N/A
EDSS score	4.2 (1–8.5)	N/A
CSF OCBs (+), *n*%	9 (23.7)	N/A
NMO‐IgG (+), *n*%	21 (55.3)	–
	15/21 NMO (71.4)	–
	2/6 Recurrent optic neuritis (33.3)	–
	3/7 Asian optic‐spinal MS (42.9)	–
	1/2 Optic neuritis associated with systemic autoimmune disease (50)	–
	0/2 Optic neuritis associated with brain lesions typical of NMO (0)	–
CSF UA (mean ± *SD*, range, μmol/L)	6.71 ± 3.17 (1.00–12.10)	4.55 ± 1.96 (1.30–9.30)
Serum UA (mean ± *SD*, range, μmol/L)	231.2 ± 79.16 (78.9–401.3)	213.0 ± 57.0 (103.4–382.5)
Clinical features, *n* (%)		
Encephalopathy	7 (18.4)	–
Brainstem	12 (31.6)	–
Cerebella	4 (10.5)	–
Pyramidal	25 (65.8)	–
Sensory	33 (86.8)	–
Visual	38 (100)	–
Myelitis	28 (73.7)	–
Sphincter disturbance	15 (39.5)	–
Number of vertebral segment spinal cord lesions seen on MRI (median, range)		
Cerebrum	1 (0–17)	
Brainstem	0 (0–3)	–
Cerebellum	0 (0–1)	–
Cervical cord	1 (0–7)	–
Thoracic cord	0 (0–9)	–

NMOSD, neuromyelitis optic spectrum disorder; NMO, neuromyelitis optic; MS, multiple sclerosis; CTLs, controls; ARR, annualized relapse rate; EDSS, expanded disability status scale; UA, uric acid; CSF, cerebrospinal fluid; OCBs, oligoclonal bands; MRI, magnetic resonance imaging; SD, standard deviation; N/A, not available.

### 2.3 Biochemical assays

Lumbar punctures were performed under standardized conditions at the L3–L4 or L4–L5 interspace. Cerebrospinal fluid samples were collected and immediately aliquoted, and frozen at −80°C until further use. Blood samples were collected at the same visit time and analyzed. Cerebrospinal fluid and serum UA levels were measured using a Clinical Analyzer 7180‐ISE (Hitachi High‐Technologies, Tokyo, Japan) according to the manufacturer's instructions.

Cerebrospinal fluid and serum albumin were quantified to calculate the CSF Albumin Index (CSF AI) as a validated marker for BBB integrity (Tibbling, Link, & Ohman, 1977). Albumin in CSF and serum was measured by routine automated laser photometry and the CSF/serum albumin ratio (CSF AI) was used to evaluate BBB disturbance (BBB index). CSF white blood cells (WBC), total protein concentration (TP), glucose (Glu), chloride (CL), NMO–immunoglobulin G (IgG), and the absence/presence of oligoclonal bands (OCB) were determined by the clinical laboratories at the Third Affiliated Hospital of Sun Yat‐sen University. The method of NMO‐IgG testing in serum was as described previously (Long et al., 2012).

### 2.4 MRI scanning

Brain, spinal cord, or optic nerve magnetic resonance imaging (MRI) scanning to detect NMOSDs was performed using a GE 1.5T MR scanner (General Electric, Milwaukee, WI, USA). The conventional MRI protocols were described in our previous paper (Zhang et al., 2014). Gadopentate dimeglumine (Gd‐DTPA) was intravenously administered at a dose of 0.1 mmol/kg, and at about 15 min after contrast injection, the T1‐weighted sequence was repeated. Patients were considered active upon MRI if there were one or more enhancing lesions in the T1‐weighted spin echo images after Gd‐DTPA injection. Lesion number and location were measured on axial sections with T2‐FLAIR sequences.

### 2.5 Statistical analysis

Data are presented as mean* *± *SD* or median with range. Differences in CSF and serum UA levels between NMOSDs and controls were analyzed by *t*‐tests. An independent *t*‐test was performed using the mean difference in CSF UA levels among subgroups of BBB integrity (intact, CSF AI < 9.0; impaired, CSF AI ≥ 9.0), gender, disease duration (≤12 months, >12 months), annualized relapse rate (ARR), disease activity, brain or spinal cord lesions upon MRI, and EDSS score. Correlations between CSF UA levels and age, serum UA levels, and CSF AI were analyzed using the Pearson test, and correlations between CSF UA levels and disease duration, EDSS score, CSF routine, and disease activity on MRI were analyzed using the Spearman's rank test. Multiple regression analysis using a stepwise method was performed to identify factors affecting CSF UA levels. A *p* < 0.05 was considered statistically significant. All statistical analyses were performed using SPSS 16.0 (SPSS Inc., Chicago, IL, USA).

## 3 Results

Patient demographic characteristics are presented in Table 1. In NMOSDs, the ratio of female to male was 29:9, the disease age of onset was 38.2 (range 17–65) years, the mean duration of disease was 23.9 (range 0.3–192) months, ARR was 0.8 (range 0.25–3.5), and mean EDSS score was 4.2 (range 1–8.5). Additionally, positive OCB in the cerebrospinal fluid was 23.7% and serum NMO‐IgG was 55.3% in the NMOSDs patients (seropositive NMO‐IgG was 71.4% [15/21] in NMO, 33.3% [2/6] in recurrent optic neuritis, 42.9% [3/7] in Asian optic‐spinal MS, 50% [1/2] in optic neuritis associated with systemic autoimmune disease, 0% [0/2] in optic neuritis associated with brain lesions typical of NMO).

**Figure 1:**
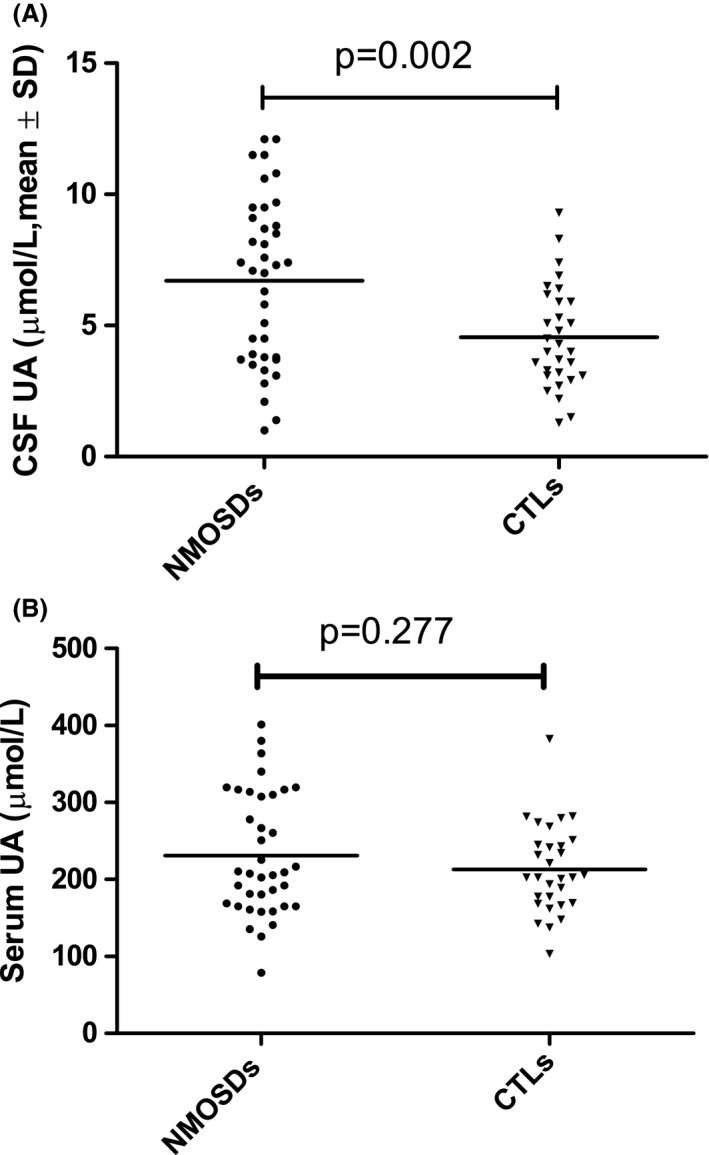
CSF UA and serum UA in NMOSDs and contols. CSF UA levels were significantly increased in the NMOSDs patients compared with CTLs (p=0.002, A). And serum UA differences between NMOSDs and CTLs were not statistically significant (p=0.277, B)

As showed in Table 1 and Fig. 1, compared with CTLs (4.55 ± 1.96 μmol/L), CSF UA levels were significantly increased in the NMOSDs patients (6.71 ± 3.17 μmol/L, *p *=* *.002) (Fig. 1A). However, there was no significant difference in serum UA levels between NMOSDs and CTLs (231.2 ± 79.16 vs., 213.0 ± 57.0 μmol/L, *p *=* *.277) (Fig. 1B).

In the present study, NMOSDs patients were further subdivided into two subgroups by gender, ARR, EDSS, disease duration, BBB integrity, disease activity upon MRI, and brain or spinal cord lesions upon MRI, as showed in Table 2. CSF UA levels in patients with longer disease durations (>12 months) were significantly higher than in those with shorter disease durations (≤1 year) (*p *=* *.002). Patients with an impaired BBB (CSF AI ≥ 9) had significantly higher CSF UA levels than those with an intact BBB (CSF AI < 9) (*p *<* *.001). Patients with MRI activity had significantly higher CSF UA levels than those with MRI inactivity (*p *<* *.001), and patients with brain lesions upon MRI had significantly higher CSF UA levels than those without brain lesions (*p *=* *.024). Additionally, CSF UA levels in male patients were higher than in female patients, CSF UA levels in severe disability patients (EDSS > 3.5) were higher than in those with mild disability (EDSS ≤ 3.5), CSF UA levels in patients with higher ARR (ARR > 1) were higher than in those with lower ARR (ARR ≤ 1), CSF UA levels in patients with longer spinal cord lesions (≥3 vertebral segments) were higher than in those with shorter spinal cord lesions (<3 vertebral segments), although these differences were not statistically significant (Table 2).

**Table 2:** CSF UA levels in patients with NMOSDs

Variables	Mean ± *SD* (μmol/L)	Range (μmol/L)	*p*
Sex			
Male (*n *= 9)	7.76 ± 3.47	1.0–12.1	
Female (*n *= 29)	6.38 ± 3.07	1.4–12.1	.261
ARR			
≤1 (*n *= 30)	6.48 ± 3.24	1.0–12.1	
>1 (*n *= 8)	7.55 ± 1.05	3.1–12.1	.404
EDSS			
≤3.5 (*n *= 19)	6.02 ± 2.85	1.0–12.1	
>3.5 (*n *= 19)	7.40 ± 3.41	1.4–12.1	.184
Disease duration			
≤12 months (*n *= 21)	5.32 ± 2.61	1.0–11.5	
>12 months (*n *= 17)	8.42 ± 3.02	1.4–12.1	**.002**
BBB			
Intact (*n *= 27)	5.31 ± 2.51	1.0–9.7	
Impaired (*n *= 11)	10.13 ± 1.65	7.3–12.1	**<.001**
MRI			
Active (*n *= 11)	9.40 ± 2.01	6.3–12.1	
Inactive (*n *= 27)	5.61 ± 2.91	1.0–11.5	**<.001**
Brain lesions			
With brain lesions (*n *= 9)	8.77 ± 3.55	1.0–12.1	
Without brain lesions (*n *= 29)	6.06 ± 2.82	1.4–11.5	**.024**
Spinal cord lesions			
<3 vertebral segments (*n *= 18)	6.52 ± 2.77	2.8–11.5	
≥3 vertebral segments (*n *= 20)	6.87 ± 3.56	1.0–12.1	.740

NMOSD, neuromyelitis optic spectrum disorder; CSF, cerebrospinal fluid; UA, uric acid; ARR, annualized relapse rate; EDSS, expanded disability status scale; MRI, magnetic resonance imaging; BBB, blood–brain barrier. *p* ‐ values that are in bold shows statistical significance.

**Figure 2:**
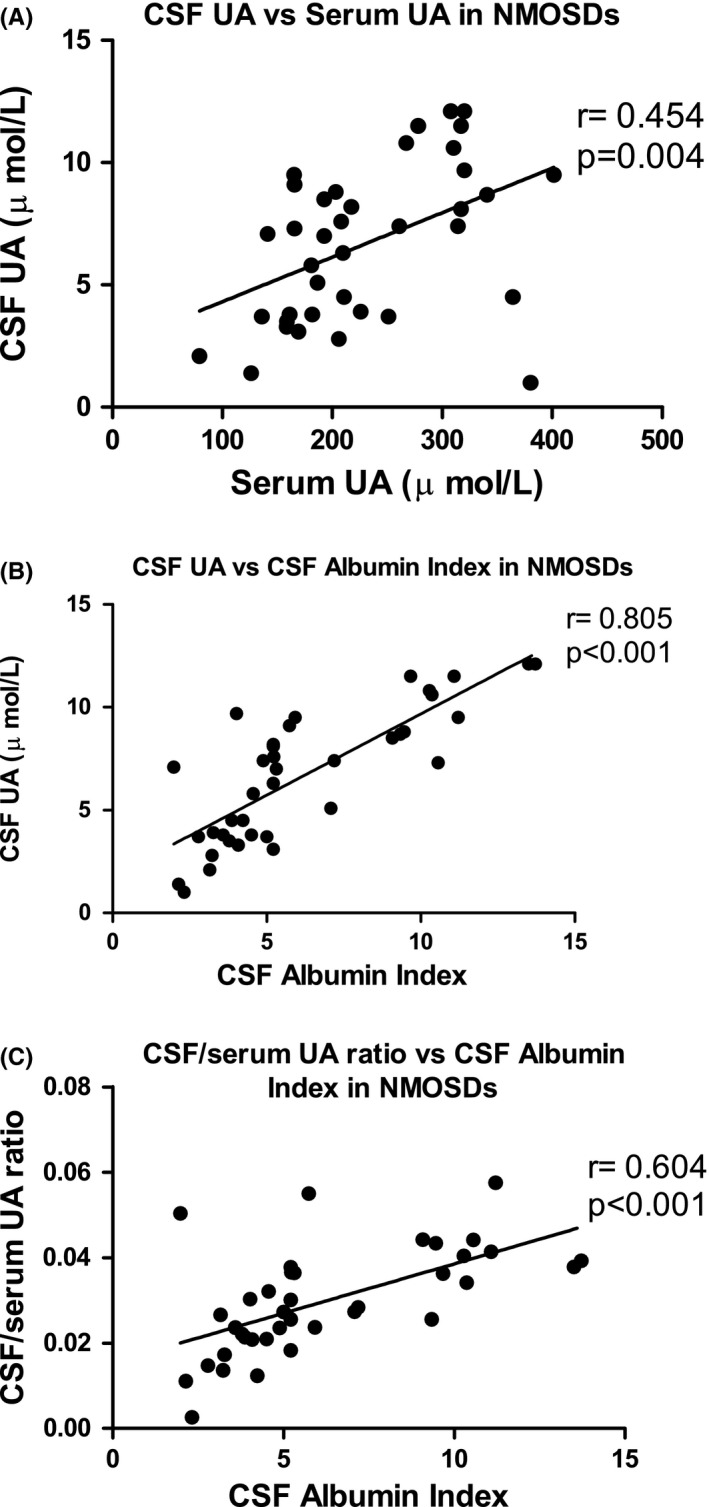
Association among CSF UA, serum UA, and BBB integrity in NMOSDs. A) Serum UA versus CSF UA, r = 0.454, p = 0.004; B) CSF UA versus CSF Albumin Index, r = 0.805, p < 0.001. C) CSF‐to‐plasma UA versus CSF Albumin Index, r = 0.604, p < 0.001

The present paper investigated the relationship between CSF UA levels and clinical characteristics, serum UA, CSF AI (BBB index), and CSF parameters in NMOSD patients (Table 3, Fig. 2). Our results showed positive correlations between CSF UA and serum UA levels (*r *= .454, *p *=* *.004) or CSF AI (*r *= .805, *p *<* *.001), and a positive correlation between CSF UA levels and disease duration (*r *= .383, *p *=* *.018). Additionally, results showed a positive correlation between CSF to plasma UA ratio and CSF AI (*r *=* *.604, *p *<* *.001). However, there were no significant correlations between CSF UA levels and age, ARR, EDSS, CSF WBC, CSF TP, CSF GLU, CSF CL, or disease activity upon MRI (Table 3).

**Table 3:** Correlation coefficients generated between CSF UA and clinical characteristics, serum uric acid, CSF parameters, and lesions on MRI in NMOSDs patients

Variable	*r*	*p*
Pearson test		
Age	−.072	.666
Serum UA	.454	**.004**
CSF Albumin Index (BBB index)	.805	**<.001**
Spearman's rank test		
Sex	.192	.248
EDSS	.122	.464
ARR	.210	.103
Disease duration	.383	**.018**
CSF WBC	−.069	.682
CSF TP	.154	.355
CSF GLU	−.252	.128
CSF CL	.074	.659
Disease activity on MRI	.289	.078

NMOSD, neuromyelitis optic spectrum disorder; CSF, cerebrospinal fluid; UA, uric acid; ARR, annualized relapse rate; EDSS, expanded disability status scale; MRI, magnetic resonance imaging; BBB, blood–brain barrier; WBC, white blood cell; TP, total protein concentration; Glu, glucose; CL, chloride. *p* ‐ values that are in bold shows statistical significance.

Multiple linear regression analysis was performed to investigate the influence of independent variables on CSF UA levels in NMOSDs patients. The dependent variable in this study was CSF UA levels in NMOSDs. The variables were compiled from clinical data (age, disease duration, ARR, EDSS, serum UA levels, CSF AI, and CSF parameters). The test collinearity diagnostics indicated that all independent variables were sufficient for the regression model. The unstandardized partial regression coefficients (β) were coefficients from the estimated regression model. The coefficient of determination (*R* ^2^) was the percentage of dependent variables explained by independent variables added to the model. Results of the multiple regression analysis are shown in Table 4. The regression analysis demonstrated continuous and linear correlations between CSF UA and serum UA levels (β = .01, *p *=* *.019), and between CSF UA levels and CSF AI (β = .72, *p *<* *.001). Serum UA levels and CSF AI together explained 69.9% (*R* ^2^ = .699) of the CSF UA variance.

**Table 4:** Multiple regression analysis models for CSF uric acid in NMOSDs patients

Variable	β (95% CI)	*p*
Serum UA	.01 (0.002, 0.017)	**.019**
CSF albumin index	.72 (0.527, 0.913)	**<.001**

β: unstandardized coefficients; CI: confidence interval; CSF: cerebrospinal fluid. *p* ‐ values that are in bold shows statistical significance.

## 4 Discussion

Results from the present study showed that UA levels in the CSF increased in NMOSD patients during clinical relapse. UA, which is the end product of adenine nucleotide catabolism, together with other oxypurines (xanthine and hypoxanthine), reflect the rate of ATP catabolism (Lazzarino et al., 2010). The ATP metabolites, including CSF UA, increased in MS patients, suggesting an increased energy demand that led to central ATP depletion (Lazzarino et al., 2010). We speculated that NMO relapse also requires greater energy demand or central ATP depletion. Because the other oxypurines (xanthine and hypoxanthine) have not been assayed, it is difficult to establish whether there was a substantial energy metabolism impairment that led to increased CSF UA levels. The correlation between serum and CSF UA levels, although statistically significant, was low (*r *= .454). Additionally, because serum UA levels in NMOSD are similar to levels found in controls, the mechanisms involved in increased CSF UA levels in NMOSD patients are not immediately clear. This small difference in serum UA might be partially responsible for the increased CSF UA, which is potentially “helped” by the imbalance of cerebral cell energy metabolism causing increased UA production.

The UA results in biological fluids from MS or NMO patients remain conflicting. Some studies have suggested that serum or CSF UA levels decrease in MS (Dujmovic et al., 2009; Peng et al., 2008; Sotgiu et al., 2002), while others show increased UA and purine compounds in biological fluids of MS patients (Amorini et al., 2009; Langemann, Kabiersch, & Newcombe, 1992; Lazzarino et al., 2010). Min et al. (2012) suggest that serum UA levels decrease in NMO patients during relapse compared with healthy subjects, but then normalize during remission, which suggests that serum UA levels are associated with clinical disease status in NMO patients. In the present paper, CSF UA levels were significantly increased, and serum UA levels were mildly elevated in NMOSD patients during relapse compared with patients with noninflammatory and non‐neurodegenerative diseases, although CSF and serum UA levels were not measured during remission. We speculated that these conflicting results might be associated with different enrolled controls, enrolled patients with different phase and sample sizes, and different assay methods.

The present study also showed that NMOSDs patients with impaired BBB, or with longer disease duration, active MRI, or brain lesions had significantly higher CSF UA levels. Oxidative stress and glutamate‐mediated excitotoxicity plays an important role in NMOSD pathogeny (Marignier et al., 2010). CSF UA levels could reflect oxidative stress and glutamate‐mediated excitotoxicity in neurological patients (Stover et al., 1997), and it has been suggested (Stover et al., 1997) that increasing oxidative stress is reflected by UA and elevated uric acid levels are associated with glutamate‐mediated excitotoxicity in CSF. Therefore, we speculated that NMOSD patients with impaired BBB, longer disease duration, active lesions upon MRI, or brain lesions suffered from greater oxidative stress and excitotoxicity.

Furthermore, CSF UA levels were dependent on CSF AI (BBB integrity, *r *= .805, *p *<* *.001) in NMOSDs patients. Recently, BBB destruction has been considered to play an important role in NMO progress (Shimizu et al., 2012). Normally, UA levels are about 20 times higher in serum than in CSF in healthy subjects (O'Connor, Harkness, Simmonds, & Hytten, 1981), but serum UA levels were about 40‐fold higher in NMOSD patients in the present study compared with CSF UA levels. Increased CSF UA levels were observed in NMOSD patients with BBB impairment, and CSF UA levels were positively associated with the BBB index. Therefore, we speculated that CSF UA might be partly produced outside the CNS, and that serum UA entering into the CNS was limited by the BBB in NMOSD patients. Interestingly, there was a positive correlation between the CSF to plasma UA ratio and the CSF albumin index in NMOSDs, suggesting that the CSF to plasma UA ratio is a marker of BBB integrity, which is consistent with previous reports (Bowman, Shannon, Frei, Kaye, & Quinn, 2010; Niklasson, Hetta, & Degrell, 1988).

In conclusion, CSF UA levels significantly increased in NMOSDs patients, and were likely modified by serum UA and BBB integrity. Results also suggested that CSF UA levels reflected oxidative stress and excitotoxicity in NMOSDs. Further studies are needed to assess the role of UA in NMOSDs.

## Conflict of Interests

The authors declare no potential conflict of interests.
